# Prognostic value of exercise-induced pulmonary congestion by lung ultrasound in patients with heart failure

**DOI:** 10.1093/eschf/xvaf025

**Published:** 2026-01-08

**Authors:** Rie Nakayama, Yoichi Takaya, Takahiro Nishihara, Rika Takemoto, Eriko Kusunoki, Yuta Ueki, Yu Yoshida, Norihisa Toh, Toru Miyoshi, Kazufumi Nakamura, Shinsuke Yuasa

**Affiliations:** Department of Cardiovascular Medicine, Dentistry and Pharmaceutical Sciences, Okayama University Graduate School of Medicine, 2-5-1 Shikata-cho, Kita-ku, Okayama 700-8558, Japan; Department of Cardiovascular Medicine, Dentistry and Pharmaceutical Sciences, Okayama University Graduate School of Medicine, 2-5-1 Shikata-cho, Kita-ku, Okayama 700-8558, Japan; Department of Cardiovascular Medicine, Dentistry and Pharmaceutical Sciences, Okayama University Graduate School of Medicine, 2-5-1 Shikata-cho, Kita-ku, Okayama 700-8558, Japan; Division of Medical Support, Okayama University Hospital, Okayama, Japan; Division of Medical Support, Okayama University Hospital, Okayama, Japan; Department of Cardiovascular Medicine, Dentistry and Pharmaceutical Sciences, Okayama University Graduate School of Medicine, 2-5-1 Shikata-cho, Kita-ku, Okayama 700-8558, Japan; Department of Cardiovascular Medicine, Dentistry and Pharmaceutical Sciences, Okayama University Graduate School of Medicine, 2-5-1 Shikata-cho, Kita-ku, Okayama 700-8558, Japan; Department of Cardiovascular Medicine, Dentistry and Pharmaceutical Sciences, Okayama University Graduate School of Medicine, 2-5-1 Shikata-cho, Kita-ku, Okayama 700-8558, Japan; Department of Cardiovascular Medicine, Dentistry and Pharmaceutical Sciences, Okayama University Graduate School of Medicine, 2-5-1 Shikata-cho, Kita-ku, Okayama 700-8558, Japan; Department of Cardiovascular Medicine, Dentistry and Pharmaceutical Sciences, Okayama University Graduate School of Medicine, 2-5-1 Shikata-cho, Kita-ku, Okayama 700-8558, Japan; Department of Cardiovascular Medicine, Dentistry and Pharmaceutical Sciences, Okayama University Graduate School of Medicine, 2-5-1 Shikata-cho, Kita-ku, Okayama 700-8558, Japan

**Keywords:** B-lines, Exercise echocardiography, Lung ultrasound, Heart failure, Pulmonary congestion

## Abstract

**Introduction:**

Pulmonary congestion during exercise is related to a poor prognosis in patients with heart failure (HF). B-Lines assessed by lung ultrasound quantitatively identify pulmonary congestion. The aim of this study was to evaluate the prognostic value of exercise-induced B-lines in patients with HF.

**Methods:**

Patients with HF who were admitted to Okayama University Hospital between February 2021 to June 2024 were enrolled. Symptom-limited exercise echocardiography using a bicycle ergometer was performed at the time of discharge. B-lines were evaluated in the 2-site simplified scan. The end point was cardiac death or hospitalization for HF.

**Results:**

A total of 124 patients with HF (71 [57–78] years, 65% male) were enrolled. The total number of B-lines was 1.9 ± 2.1 at rest, which significantly increased to 5.4 ± 3.5 at the peak workload of the average 40 W. In the receiver operating characteristic curve, the cut-off value of exercise-induced B-lines ≥7 at peak workload was related to cardiac events (sensitivity: 50%, specificity: 79%). During the follow-up period of 25 months, 26 patients had cardiac events. Kaplan–Meier analysis showed that the event-free survival rate was significantly worse in patients with B-lines ≥7 at peak workload than in those with B-lines <7 at peak workload (log-rank test, *P* = .002). B-lines ≥7 at peak workload were independently related to cardiac events.

**Conclusion:**

Exercise-induced B-lines are associated with cardiac death or hospitalization for HF in patients with HF. B-lines at peak workload may identify risk stratification of HF.

## Graphical Abstract

**Graphical Abstract:**
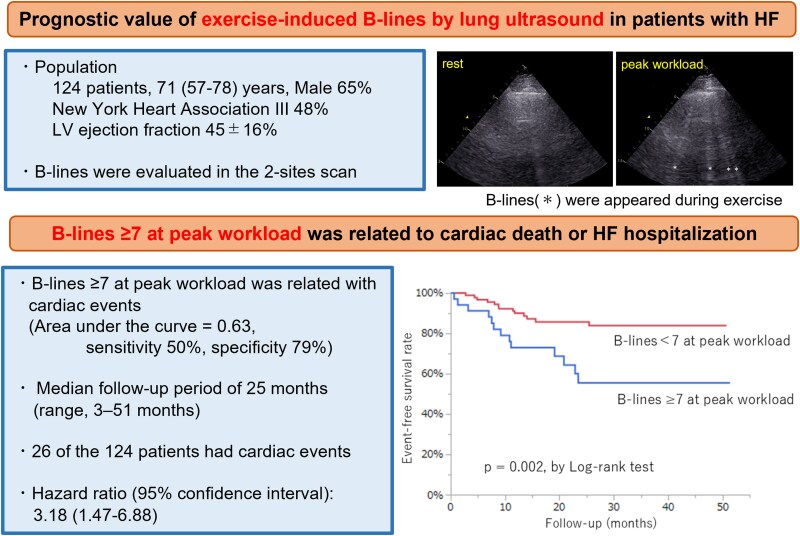
B-lines in heart failure

## Introduction

Heart failure (HF) leads to mortality at a high rate. Pulmonary congestion is associated with a poor prognosis in patients with HF.^1–3^ B-lines assessed by lung ultrasound, which are known as ultrasound lung comets, are a reliable and reproducible tool for quantitatively identifying pulmonary congestion.^4,5^ The presence of resting B-lines has been demonstrated to be associated with clinical outcomes.^3,6,7^

Patients with HF develop dyspnoea upon exercise. Exercise stress is valuable for assessing changes of pulmonary congestion.^8,9^ In recent studies, an increase in B-lines during exercise has been reported to be useful for predicting cardiac events.^10–12^ However, limited information is available in patients with HF. The efficacy of exercise-induced B-lines on clinical outcomes remains unknown in patients with advanced HF, for whom risk stratification is important for therapeutic management of HF.

We hypothesized that B-lines during exercise sensitively predict adverse outcomes in patients with an advanced stage of HF. This study aimed to evaluate the prognostic value of exercise-induced B-lines by lung ultrasound in patients hospitalized for HF.

## Methods

### Study population

We prospectively enrolled 124 patients with HF who were admitted to Okayama University Hospital from February 2021 to June 2024 and underwent exercise echocardiography at the time of discharge. The diagnosis of HF was made based on clinical findings, including symptoms, physical examination, electrocardiogram, echocardiography, biomarker measurements, and chest X-ray, according to the guidelines of the European Society of Cardiology.^13^ Patients with a history or signs of lung disease, such as interstitial pneumonia, obstructive pulmonary disease, and/or lung cancer, were excluded. This study was performed according to the principles of the Declaration of Helsinki and was approved by the ethics committee of our institution. Informed consent was obtained before participating in the study.

### Clinical parameters

Clinical characteristics, including age, sex, laboratory data, and medical therapy, were obtained from medical records. Transthoracic echocardiographic parameters, such as left ventricular (LV) end-diastolic and end-systolic volumes, LV ejection fraction, LV mass index, left atrial (LA) volume index, early diastolic mitral inflow velocity (*E*), early diastolic mitral annular velocity (*e*’), early diastolic mitral inflow velocity to mitral annular velocity ratio (*E*/*e*’), tricuspid regurgitation (TR) pressure gradient, and inferior vena cava diameter, were measured according to the guidelines from the American Society of Echocardiography.^14^ LV ejection fraction was calculated using the disk summation method.

### Exercise echocardiography

Symptom-limited exercise echocardiography using a bicycle ergometer was performed in a supine, slightly left lateral decubitus position at the time of discharge from HF treatment. Exercise started at an initial workload of 20 W and increased stepwise by 10 or 20 W every 2 min until exhaustion while maintaining a pedalling speed of 60 rpm. Electrocardiogram and oxygen saturation were monitored continuously. Blood pressure and heart rate were recorded every 2 min. Exercise was interrupted in the event of chest pain, ST-segment shift, excessive blood pressure increase (systolic blood pressure, ≥240 mmHg; diastolic blood pressure, ≥120 mmHg), limiting dyspnoea, or significant arrhythmias.

Transthoracic echocardiography was performed at rest and during exercise. Two-dimensional and Doppler echocardiographic images were obtained. *E*, *e*’, *E*/*e*’, and TR pressure gradient were measured at rest and at peak workload.

### Lung ultrasound

**Figure 1:**
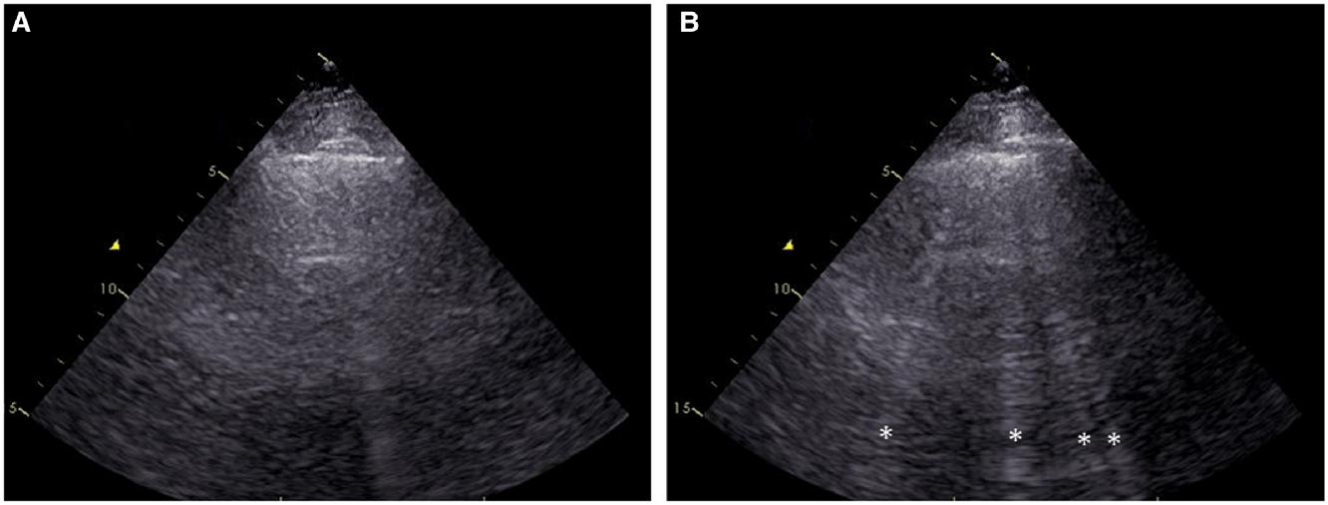
The number of B-lines was evaluated by lung ultrasound at rest (A) and at peak workload (B). Exercise-induced B-lines appeared (*)

Lung ultrasound was performed using the same cardiac transducer as that for transthoracic echocardiography. During exercise echocardiography using a bicycle ergometer, lung ultrasound was performed at rest and at peak workload. We adopted the two-site simplified scan. B-lines were evaluated online in the two sites, with one site on the left side and one on the right side, from parasternal to anterior axillary lines on the third and fourth intercostal spaces. B-Lines were defined as a discrete, laser-like vertical hyperechoic reverberation artefact arising from the pleural line, extending to the bottom of the screen without fading, and moving synchronously with lung sliding. The total number of B-lines in the two sites was calculated at rest and at peak workload (*Figure 1*). All examinations were performed by a single operator who was blinded to patients’ data and did not take part in clinical management.

### Endpoint

The endpoint was cardiac death or hospitalization for HF. Patients were followed from the date of exercise echocardiography until the date of first documentation of cardiac events or the end of follow-up. Follow-up data were obtained from medical records.

### Statistical analysis

Data are presented as mean ± standard deviation or median (interquartile range) for continuous variables and as number and percentage for categorical variables. Continuous variables were compared using the *t*-test or Mann–Whitney *U* test. Categorical variables were compared using the χ*^2^* test. Correlations between B-lines and echocardiographic parameters were assessed. Pearson correlation coefficients were calculated for B-lines at rest and at peak workload with LA volume index, *E*/*e*′, and TR pressure gradient. The cut-off value of B-lines for the relationship with cardiac events was estimated using a receiver operating characteristic curve. Event-free survival rate was estimated using the Kaplan–Meier analysis, with group differences compared using the log-rank test. Cox proportional hazard regression analysis was performed to identify independent factors related to cardiac events. Variables for analysis included age, sex, body mass index, New York Heart Association class, N-terminal proBNP natriuretic peptide (NT-proBNP) level, LV ejection fraction, LA volume index, *E*/*e*’, TR pressure gradient, and B-lines. Hazard ratios are shown with 95% confidence intervals. Statistical analysis was performed with statistical software (JMP version 14.0; SAS Institute Inc., Cary, NC, USA), and significance was defined as a *P* value of <.05.

Interobserver and intra-observer differences were analysed for 20 stored images selected randomly. The number of B-lines was evaluated by two blinded observers and by a single observer at two different time points, both at rest and at peak workload. The number of B-lines was counted directly from the images. Reliability was calculated using Pearson’s correlation coefficient. Variability was calculated as the percentage error of each measurement, defined as the absolute difference between the two measurements divided by their mean value and expressed as a percentage.

## Results

### Patient characteristics

Patient characteristics are shown in *Table 1*. The median age of all patients was 71 (57–78) years, and 81 patients were male. Fifty-four patients had HF with reduced ejection fraction, 21 had HF with mildly reduced ejection fraction, and 49 had HF with preserved ejection fraction. Sixty patients had New York Heart Association Class III. The mean LV ejection fraction was 45 ± 16%. The mean *E*/*e*’ was 19 ± 11, and the mean TR pressure gradient was 22 ± 9 mmHg. Many patients received beta-blockers and diuretics or tolvaptan. When patients were divided according to B-lines at peak workload, patients with B-lines ≥7 at peak workload were older and had a higher prevalence of atrial fibrillation and worse renal function. There was no significant difference in New York Heart Association class between the two groups. Medical therapies were similar between the groups, except for a higher use of loop diuretics in patients with B-lines ≥7 at peak workload.

**Table 1:** Clinical characteristics

Variables	All (*n* = 124)	B-line ≥7 at peak workload (*n* = 34)	B-lines <7 at peak workload (*n* = 90)	*P*-value
**Age, years**	71 (57–78)	76 (68–80)	69 (54–76)	<.01
**Male, *n* (%)**	81 (65)	25 (74)	56 (62)	.23
**Body mass index, kg/m^2^**	23 ± 4	23 ± 4	23 ± 5	.66
**Hypertension, *n* (%)**	42 (34)	16 (47)	26 (29)	0.06
**Diabetes mellitus, *n* (%)**	28 (23)	9 (26)	19 (21)	.52
**Dyslipidaemia, *n* (%)**	39 (31)	15 (44)	24 (27)	.06
**Ischaemic heart disease, *n* (%)**	21 (17)	8 (24)	13 (14)	.23
**Atrial fibrillation, *n* (%)**	25 (20)	13 (38)	12 (13)	<.01
**HF with reduced ejection fraction, *n* (%)**	54 (44)	18 (53)	36 (40)	.19
**HF with mildly reduced ejection fraction, *n* (%)**	21(17)	5 (15)	16 (18)	.68
**HF with preserved ejection fraction, *n* (%)**	49 (40)	11 (32)	38 (42)	.32
**New York Heart Association Class I/II/III, *n* (%)**	4/60/60 (3/48/48)	0/14/20 (0/41/59)	4/46/40 (4/51/44)	.22
**Laboratory data**				
** NT-proBNP, pg/mL (*n* = 60)**	890 (339–1950)	1820 (1166–3526)	748 (267–1549)	.04
** Creatinine, mg/dL**	1.2 ± 0.5	1.4 ± 0.5	1.1 ± 0.5	<.01
** Estimated glomerular filtration rate, mL/min/1.73m^2^**	52 ± 24	41 ± 18	56 ± 24	<.01
** Sodium, mEq/L**	139 ± 3	138 ± 3	139 ± 3	.32
**Echocardiographic measurements**				
** LV end-diastolic volume, ml**	132 ± 59	135 ± 65	131 ± 57	.73
** LV end-systolic volume, ml**	80 ± 54	82 ± 56	79 ± 54	.75
** LV ejection fraction, %**	45 ± 16	43 ± 15	45 ± 16	.53
** LV mass index, g/m^2^**	122 ± 33	128 ± 35	120 ± 32	.24
** LA volume index, ml/m^2^**	57 ± 26	64 ± 26	54 ± 26	.04
** *E*, cm/s**	92 ± 41	106 ± 36	87 ± 41	.02
** *e*’, cm/s**	5.3 ± 2.5	5.2 ± 1.6	5.4 ± 2.7	.78
** *E/e*’**	19.4 ± 10.9	21.4 ± 8.4	18.7 ± 11.7	0.23
**Mitral regurgitation** **none/mild/moderate/severe, *n* (%)**	44/37/35/8 (35/30/28/6)	7/7/15/5 (21/21/44/15)	37/30/20/3 (41/33/22/3)	<.01
**TR none/mild/moderate/severe, *n* (%)**	56/31/24/13 (45/25/19/10)	10/12/5/7 (29/35/15/21)	46/19/19/6 (51/21/21/7)	.02
**TR pressure gradient, mmHg**	25 ± 11	27 ± 11	25 ± 11	.24
**Inferior vena cava diameter, mm**	13 ± 6	14 ± 6	13 ± 5	.30
**Medical therapy**				
** Angiotensin-converting enzyme inhibitors, angiotensin receptor blockers, or angiotensin receptor blockers, *n* (%)**	87 (70)	25 (74)	62 (69)	.61
** Beta-blockers, *n* (%)**	106 (85)	30 (88)	76 (84)	.59
** Aldosterone antagonists, *n* (%)**	95 (77)	26 (76)	69 (77)	.98
** Sodium-glucose transporter 2 inhibitors, *n* (%)**	80 (65)	23 (68)	57 (63)	.65
** Loop diuretics, (%)**	69 (56)	24 (71)	45 (50)	.02
** Loop diuretic dose, mg**	26 ± 18	30 ± 19	25 ± 17	.25
** Tolvaptan, *n* (%)**	24 (19)	9 (26)	15 (17)	.22

*E*, early diastolic mitral inflow velocity; *e*’, early diastolic mitral annular velocity; *E*/*e*’, early diastolic mitral inflow velocity to mitral annular velocity ratio; HF, heart failure; LA, left atrial; LV, left ventricular; NT-proBNP, N-terminal pro-B-type natriuretic peptide; TR, tricuspid regurgitation.

Data are presented as mean ± standard deviation, median (interquartile range), or number (%) of patients.

### Exercise echocardiography and lung ultrasound

Exercise echocardiography and lung ultrasound measurements are summarized in *Table 2*. The average achieved peak workload was 40 W (range, 20–120 W). TR pressure gradient and *E*/*e*’ were significantly increased at peak workload compared to those at rest. The total number of B-lines was 1.9 ± 2.1 at rest, which significantly increased to 5.4 ± 3.5 at peak workload. The assessment of B-lines by lung ultrasound was feasible in all patients.

**Table 2:** Exercise echocardiography and lung ultrasound measurements at rest and at peak workload

Variables	Rest	Peak workload	*P*-value
**Systolic blood pressure, mmHg**	119 ± 22	138 ± 28	<.01
**Diastolic blood pressure, mmHg**	64 ± 13	75 ± 18	<.01
**Heart rate, bpm**	69 ± 12	93 ± 22	<.01
**Oxygen saturation, %**	98 ± 2	96 ± 2	<.01
***E*, cm/s**	92 ± 41	113 ± 44	<.01
***e*’, cm/s**	5.3 ± 2.5	6.3 ± 3.0	<.01
***E*/*e*’**	19 ± 11	21 ± 12	.04
**TR pressure gradient, mmHg**	22 ± 9	36 ± 16	<.01
**B-lines**	1.9 ± 2.1	5.4 ± 3.5	<.01

*E*, early diastolic mitral inflow velocity; *e*’, early diastolic mitral annular velocity; *E*/*e*’, early diastolic mitral inflow velocity to mitral annular velocity ratio; TR, tricuspid regurgitation.

Data are presented as mean ± standard deviation.

**Figure 2:**
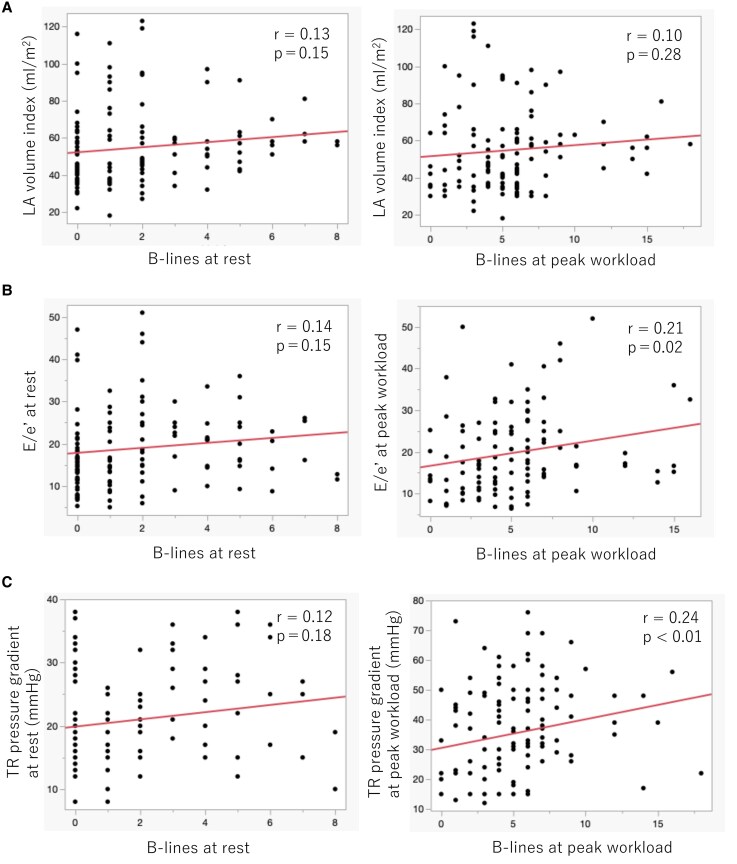
Correlations between B-lines and echocardiographic parameters. Scatter plots demonstrated the correlations of B-lines with LA volume index (A), *E*/*e*′ (B), and TR pressure gradient (C). There were significant correlations of B-lines at peak workload with *E*/*e*′ at peak workload (*r* = 0.21, *P* = .02) and TR pressure gradient at peak workload (*r* = 0.24, *P* < .01). *E*/*e*′, early diastolic mitral inflow velocity to mitral annular velocity ratio; LA, left atrial; TR, tricuspid regurgitation

There were significant correlations of B-lines at peak workload with *E*/*e*′ at peak workload (*r* = 0.21, *P* = .02) and TR pressure gradient at peak workload (*r* = 0.24, *P* < .01). B-lines did not correlate with LA volume index (*Figure 2*).

### B-Lines and cardiac events

**Figure 3:**
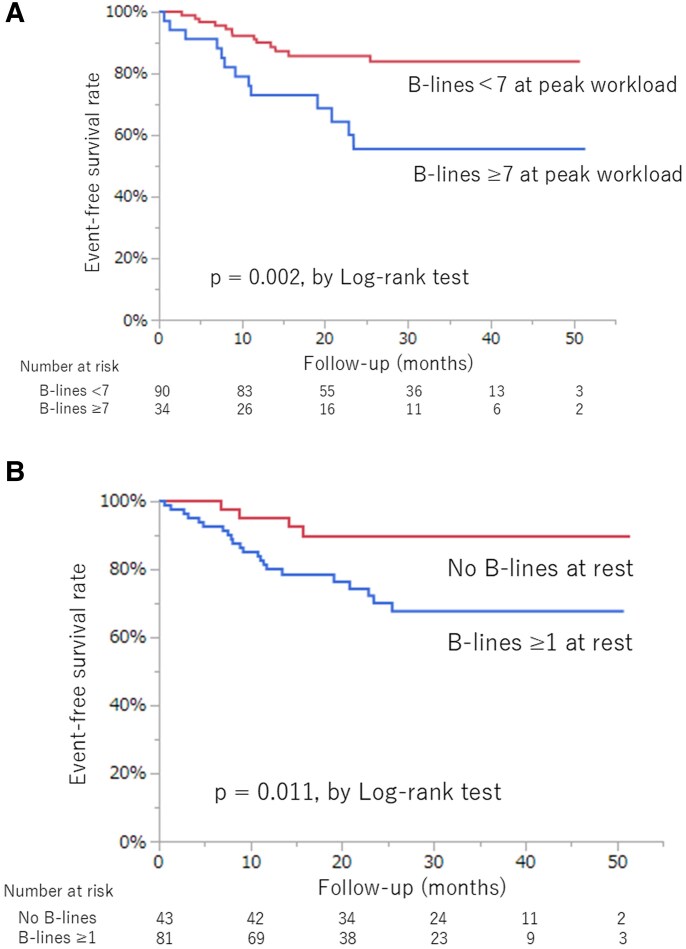
Event-free survival rate according to the total number of B-lines at rest and at peak workload. (A) The event-free survival rate was worse in patients with B-lines ≥7 at peak workload than in those with B-lines <7. (B) The event-free survival rate was worse in patients with B-lines ≥1 at rest than in those without B-lines

During the median follow-up period of 25 months (range, 3–51 months), 26 of the 124 patients had cardiac events. The optimal cut-off value of the total number of B-lines at peak workload for the relationship with cardiac events was ≥7 (area under the curve = 0.63), with a sensitivity of 50% and specificity of 79%. Of the 34 patients with B-lines ≥7 at peak workload, 2 had cardiac death and 11 were hospitalized for HF. Of the 90 patients with B-lines <7 at peak workload, 1 died and 12 were hospitalized for HF. Kaplan–Meier analysis showed that the event-free survival rate was significantly worse in patients with B-lines ≥7 at peak workload than in those with B-lines <7 (log-rank test, *P* = .002) (*Figure 3A*).

The optimal cut-off value of B-lines at rest for the relationship with cardiac events was ≥1 (area under the curve = 0.62), with a sensitivity of 84% and specificity of 40%. Kaplan–Meier analysis showed that the event-free survival rate was worse in patients with B-line ≥1 at rest than in those without B-lines (log-rank test, *P* = .011) (*Figure 3B*).

In the Cox proportional hazards analysis, univariable analysis showed that age, LA volume index, *E*/*e*’ at rest, *E*/*e*’ at peak workload, B-lines at rest, and B-lines at peak workload were related to cardiac events. Multivariable analysis demonstrated that B-lines ≥7 at peak workload were independently related to cardiac events in models 1, 2, 3, and 4. When NT-proBNP level and B-lines at peak workload were included in multivariate analysis of model 5, B-lines at peak workload tended to be related to cardiac events (*Table 3*).

**Table 3:** Factors related to cardiac death or heart failure hospitalization

Variables	Univariate analysis	*P*	Multivariate analysis 1	*P*	Multivariate analysis 2	*P*	Multivariate analysis 3	*P*	Multivariate analysis 4	*P*	Multivariate analysis 5	*P*
	Hazard ratio (95% confidence interval)		Hazard ratio (95% confidence interval)		Hazard ratio (95% confidence interval)		Hazard ratio (95% confidence interval)		Hazard ratio (95% confidence interval)		Hazard ratio (95% confidence interval)	
**Age ≥60 years**	3.54 (1.06–11.8)	.04	2.76 (0.78–9.71)	.11								
**Male**	1.96 (0.78–4.88)	.15	1.69 (0.65–4.38)	.28								
**Body mass index ≥25 kg/m^2^**	1.25 (0.57–2.72)	.57	1.17 (0.52–2.64)	.70								
**New York Heart Association Class III**	1.65 (0.75–3.65)	.21										
**NT-proBNP ≥300 pg/mL**	2.97 (0.38–23.5)	.30									2.45 (0.30–19.7)	.40
**LV ejection fraction <40%**	0.96 (0.44–2.10)	.92										
**LA volume index ≥52 ml/m^2^**	2.67 (1.16–6.15)	.02			2.00 (0.78–5.17)	.15						
***E*/*e*’ ≥14 at rest**	3.98 (1.19–13.3)	.02			1.89 (0.43–8.26)	.40						
***E*/*e*’ ≥14 at peak workload**	5.99 (1.40–25.5)	.02					4.52 (1.02–19.9)	.02				
**TR pressure gradient ≥35 mmHg at rest**	0.46 (0.06–3.42)	.45										
**TR pressure gradient ≥50 mmHg at peak workload**	0.70 (0.24–2.02)	.51							0.59 (0.20–1.74)	.34		
**B-lines ≥1 at rest**	3.69 (1.26–10.7)	.02										
**B-lines ≥7 at peak workload**	3.18 (1.47–6.88)	<.01	2.31 (1.03–5.17)	.04	2.50 (1.07–5.81)	.03	2.31 (1.02–5.27)	.04	3.33 (1.54–7.23)	<.01	3.56 (0.97–13.1)	.06

*E*/*e*’, early diastolic mitral inflow velocity to mitral annular velocity ratio; LA, left atrial; LV, left ventricular; NT-proBNP, N-terminal pro-B-type natriuretic peptide; TR, tricuspid regurgitation.

Variables for multivariate analysis 1 included age, sex, body mass index, and B-lines at peak workload. Variables for multivariate analysis 2 included LA volume index, *E*/*e*’ at rest, and B-lines at peak workload. Variables for multivariate analysis 3 included *E/e*’ at peak workload and B-lines at peak workload. Variables for multivariate analysis 4 included TR pressure gradient at peak workload and B-lines at peak workload. Variables for multivariate analysis 5 included NT-proBNP level and B-lines at peak workload.

### Changes in B-lines

The median value of the difference in B-lines between rest and peak workload (ΔB-lines) was 4. When patients were divided according to ΔB-lines of 4, Kaplan–Meier analysis showed that the event-free survival rate was not different between patients with ΔB-lines ≥4 and those with ΔB-lines <4 (Supplementary data online, *Fig. S1*).

### Left ventricular ejection fraction phenotype

When patients were divided into two groups according to LV ejection fraction phenotype, B-lines at rest and at peak workload, ΔB-lines, *E*/*e*’, TR pressure gradient, LA volume index, or NT-proBNP level were not different between patients with HF with preserved ejection fraction and those with HF with reduced and mildly reduced ejection fraction (Supplementary data online, *Table S1*).

### Reproducibility

There was good agreement in the measurements of B-lines counts between the two blinded observers at rest (*r* = 0.95, *P* < .01) and between measurements obtained by the same observer at two different time points at rest (*r* = 0.96, *P* < .01). Similarly, there was good agreement for B-lines counts obtained at peak workload between the two blinded observers (*r* = 0.92, *P* < .01) and between measurements obtained by the same observer at two different time points at peak workload (*r* = 0.94, *P* < .01).

## Discussion

The major findings of the present study are as follows: (i) patients with the total number of B-lines ≥7 at peak workload in the 2-site simplified scan were more likely to have cardiac death or hospitalization for HF, and (ii) B-lines at peak workload independently predicted adverse outcomes. Our findings suggest that exercise-induced B-lines could be useful for risk stratification of HF. This study is the first to evaluate the prognostic value of exercise-induced B-lines simply assessed by lung ultrasound in patients with HF.

### Lung ultrasound

Lung ultrasound is an easy, fast, and reliable tool for evaluating pulmonary congestion.^4,5,15^ Multiple comet-tails fanning out from the lung surface due to water-thickened or fibrotic interlobular septa, called B-lines.^16^ B-lines directly image extravascular lung water.^17,18^ Several studies have demonstrated that the presence of B-lines by lung ultrasound has diagnostic and prognostic meaning in patients with HF.^3,6,7^ Additionally, B-lines are useful as a marker of HF therapeutic management. Randomized studies have demonstrated that diuretic therapy guided by B-lines is effective in reducing hospitalization in patients with HF.^19–21^

### Exercise-induced B-lines

Exercise causes an increase in pulmonary capillary wedge pressure, leading to water transfer from the vascular to the extravascular compartment. Exercise stress is valuable for assessing dynamic changes of pulmonary congestion.^11,22^ B-lines assessed by lung ultrasound develop upon exercise, which is correlated with high pulmonary capillary wedge pressure.^23^

Although a few studies have demonstrated the utility of exercise-induced B-lines for predicting clinical outcomes, limited information is available. Coiro *et al*.^10^ reported that B-lines during exercise were a predictor of a composite outcome of cardiovascular death or HF hospitalization at 1 year in 61 patients with HF preserved ejection fraction with New York Heart Association Class I or II. Scali *et al*.^12^ reported that exercise-induced B-lines were associated with a composite outcome of death, HF hospitalization, or myocardial infarction in 103 patients with HF reduced ejection fraction. However, these previous studies included patients with relatively mild HF symptoms. In the previous study of HF reduced ejection fraction, more than two-thirds of the subjects were outpatients, and many patients had New York Heart Association Class I or II.^12^ Therefore, the efficacy of exercise-induced B-lines on clinical outcomes remains unclear in patients with an advanced stage of HF.

The present study focused on the population of patients hospitalized for HF, who had HF stage C or D. Most patients had New York Heart Association Class II or III. For such patients, the prognostic stratification is important to prevent re-hospitalization for HF in the near future, and is useful for more careful monitoring and tailored treatment for HF.^24^

### Present study

The present study showed that the total number of exercise-induced B-lines ≥7 at peak workload in the two sites of the right and left sides was related to cardiac death or hospitalization for HF. Exercise was performed using a bicycle ergometer in the supine position, which allows real-time assessment of pulmonary congestion by lung ultrasound. Exercise-induced B-lines were measured at the time of maximal workload during exercise, but not after the end of exercise. This allowed for accurate evaluation of exercise-induced pulmonary congestion.

Lung ultrasound is usually measured in the 28-site scan. Recently, some studies have used the four-site simplified scan for evaluating B-lines during exercise.^11^ One study reported that the four-site scan saves time and provides accurate information.^25^ Small acquisition size of one- or two-site scan has also been proposed, although further experience is needed to confirm the accuracy. In the present study, we used the two-site simplified scan, with one site on the left side and one on the right side, because of the time constraints for imaging during exercise. Especially in patients with HF, it is difficult to maintain maximal workload for performing various measurements using transthoracic echocardiography and lung ultrasound. The two-site simplified scan is able to count B-lines at maximal workload in a short time. This technique has the potential to be widely used in the clinical setting.

The present study quantified B-lines at peak workload. As the cut-off value, we selected the total number of B-lines ≥7 at peak workload in the two sites. This grade seems to be comparable with the range of moderate to severe levels of B-lines showing in the previous report, in which the B-lines score was categorized into four levels, such as absent, mild, moderate, and severe.^26^ B-lines at peak workload weakly correlated with *E*/*e*’ at peak workload and TR pressure gradient at peak workload, whereas there was no correlation of B-lines with LA volume index indicated chronic diastolic burden. This finding suggests that B-lines may reflect dynamic pulmonary congestion beyond echocardiographic diastolic parameters.

Exercise-induced pulmonary hypertension has been reported to be a predictor of adverse outcomes.^10,27^ In the present study, while B-lines were significantly related to cardiac events, no such relationship was observed for TR pressure gradient. The reasons for this divergence warrant further investigation, but it suggests that exercise-induced extravascular lung water accumulation, as reflected by B-lines, may be a more sensitive and direct indicator of impending cardiac events in congestive HF. This could be because B-lines directly reflect the functional consequence of increased pulmonary capillary wedge pressure, leading to fluid transudation in a dynamic exercise setting, which might precede or be more critically linked to clinical decompensation.

The difference in B-lines between rest and peak workload reflects exercise-induced changes in pulmonary congestion. In the present study, ΔB-lines were not related to cardiac death or hospitalization for HF. Some previous studies reported that the absolute number of B-lines was associated with adverse outcomes.^10,12^ The prognostic value of ΔB-lines remains unknown. Further studies are required to evaluate the effect of changes in B-lines on clinical outcomes.

### Clinical implication

The present study demonstrated the utility of quantifying B-lines at peak workload, simply assessed by lung ultrasound of the two-site simplified scan, for predicting HF prognosis. Because lung ultrasound is easy to interpret, the criterion of pulmonary congestion is likely to be widely used for identifying the severity of HF in clinical practice. Additionally, therapeutic management of congestion is important to improve clinical outcomes, including the prevention of HF hospitalization. Exercise-induced B-lines might have a role in guiding HF treatment and determining their effectiveness.

### Study limitations

There are several limitations in the present study. First, this was a single-center study. The number of patients was small, and the length of follow-up was short. Because this study included patients with HF of various aetiologies, the criteria did not specifically focus on congestion. Second, there was selection bias because patients who could perform exercise echocardiography were enrolled. This study did not include patients with severe HF symptoms who were unable to exercise. Third, invasive haemodynamic assessments were not performed. Fourth, this study evaluated B-lines in the two-site simplified scan. The number of B-lines might change depending on the lung compartment. Fifth, the cut-off value of B-lines ≥7 at peak workload had moderate predictive ability and low sensitivity. B-lines ≥1 at rest, which had low specificity, might not be a pathological sign. Further studies are needed to clarify the cut-off value of B-lines. Sixth, this study included both patients with HF with reduced ejection fraction and those with HF with preserved ejection fraction. Because the number of B-lines was measured only at peak workload, dynamic congestion might be underestimated in patients with HF with preserved ejection fraction, having the peak at the recovery phase. Finally, because this study had few events, multivariate analysis was limited. The level of creatinine, estimated glomerular filtration rate, or sodium was not included in the multivariate analysis.

## Conclusion

Exercise-induced B-lines are associated with cardiac death or hospitalization for HF in patients with HF. B-lines assessment during exercise may identify risk stratification of HF. Exercise-induced B-lines may serve as a valuable and easily obtainable therapeutic indicator to manage congestion in patients with HF.

## Supplementary Material

xvaf025_Supplementary_Data

## Data Availability

No data were generated or analysed for this manuscript.

## Pre-registered Clinical Trial Number

None supplied.
