# Supplementary figures and images for: Prognostic value of exercise-induced pulmonary congestion by lung ultrasound in patients with heart failure
Source: ESC Heart Fail. 2026 Jan 8;13(1):xvaf025. doi: 10.1093/eschf/xvaf025 (PMC13108275; doi:10.1093/eschf/xvaf025)

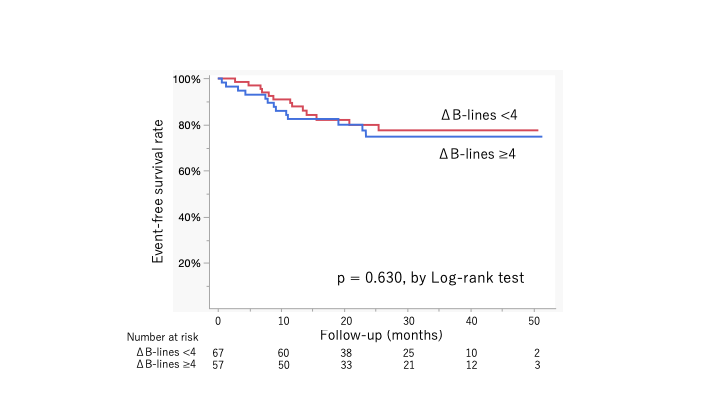

Supplement: xvaf025_Supplementary_Data [file xvaf025_supplementary_data.zip › Supplementary Figure.tiff]
