# Supplementary material for: Prognostic value of exercise-induced pulmonary congestion by lung ultrasound in patients with heart failure
Source: ESC Heart Fail. 2026 Jan 8;13(1):xvaf025. doi: 10.1093/eschf/xvaf025 (PMC13108275; doi:10.1093/eschf/xvaf025)
Supplement: xvaf025_Supplementary_Data [file xvaf025_supplementary_data.zip › Supplementary Table. rev doc..doc]

| Supplementary Table 1. Clinical characteristics according to LV ejection fraction phenotype | | | |
| --- | --- | --- | --- |
| Variables | HF with preserved ejection fraction  (n = 49) | HF with reduced and mildly-reduced ejection fraction  (n = 75) | p |
| Age, years | 69 (57-81) | 75 (55-76) | 0.04 |
| Male, n (%) | 29 (59) | 52 (69) | 0.25 |
| Body mass index, kg/m^2^ | 23 ± 4 | 23 ± 4 | 0.84 |
| NT-proBNP, pg/mL | 638 (179-1625) | 1247 (670-2681) | 0.32 |
| LA volume index, ml/m^2^ | 62 ± 33 | 53 ± 20 | 0.08 |
| E/e' at rest | 21 ± 13 | 19 ± 9 | 0.32 |
| E/e' at peak workload | 21 ± 13 | 20 ± 10 | 0.68 |
| TR pressure gradient at rest, mmHg | 23 ± 9 | 21 ± 8 | 0.11 |
| TR pressure gradient at peak workload, mmHg | 39 ± 19 | 35 ± 14 | 0.15 |
| B-lines at rest | 1.8 ± 2.1 | 1.9 ± 2.1 | 0.72 |
| B-lines at peak workload | 5.0 ± 3.3 | 5.6 ± 3.7 | 0.37 |
| ΔB-lines | 3.2 ± 2.2 | 3.7 ± 2.4 | 0.30 |
| Data are presented as mean ± standard deviation, median (interquartile range), or number (%) of patients.  E/e’ = early diastolic mitral inflow velocity to mitral annular velocity ratio, HF = heart failure, LA = left atrial, LV = left ventricular, NT-proBNP = N-terminal pro-B-type natriuretic peptide, TR = tricuspid regurgitation. | | | |
